# Nevirapine-Based Antiretroviral Therapy Impacts Artesunate and Dihydroartemisinin Disposition in HIV-Infected Nigerian Adults

**DOI:** 10.1155/2012/703604

**Published:** 2012-03-05

**Authors:** Fatai A. Fehintola, Kimberly K. Scarsi, Qing Ma, Sunil Parikh, Gene D. Morse, Babafemi Taiwo, Ibrahim Tope Akinola, Isaac F. Adewole, Niklas Lindegardh, Aphiradee Phakderaj, Oladosu Ojengbede, Robert L. Murphy, Olusegun O. Akinyinka, Francesca T. Aweeka

**Affiliations:** ^1^Department of Clinical Pharmacology, University College Hospital and Department of Pharmacology & Therapeutics, College of Medicine, University of Ibadan, Ibadan, Nigeria; ^2^Division of Infectious Diseases & Center for Global Health, Northwestern University Feinberg School of Medicine, Chicago, IL 60614, USA; ^3^Translational Pharmacology Research Core, NYS Center of Excellence in Bioinformatics and Life Sciences, Department of Pharmacy Practice, School of Pharmacy and Pharmaceutical Sciences, University at Buffalo, Buffalo, NY 14203, USA; ^4^Department of Medicine, San Francisco General Hospital, University of California, San Francisco, San Francisco, CA 94110, USA; ^5^Department of Obstetrics & Gynecology, University College Hospital, Ibadan, Nigeria; ^6^Department of Obstetrics & Gynecology, College of Medicine, University of Ibadan, Ibadan, Nigeria; ^7^Mahidol-Oxford Tropical Medicine Research Unit, Faculty of Tropical Medicine, Mahidol University, Bangkok, Thailand; ^8^Department of Pediatrics, College of Medicine, University of Ibadan, Ibadan, Nigeria; ^9^Drug Research Unit, Department of Clinical Pharmacy, School of Pharmacy, University of California, San Francisco, San Francisco, CA 94110, USA

## Abstract

*Background*. Nevirapine- (NVP-) based antiretroviral therapy (ART) and artesunate-amodiaquine are frequently coprescribed in areas of HIV and malaria endemicity. We explored the impact of this practice on artesunate and dihydroartemisinin pharmacokinetics. *Methods*. We conducted a parallel-group pharmacokinetic comparison between HIV-infected patients receiving NVP-based ART (*n* = 10) and ART-naive controls (*n* = 11). Artesunate-amodiaquine 200/600 mg was given daily for three days. Measurement of drug concentrations occurred between 0 and 96 hours after the final dose. Pharmacokinetic parameters were determined using noncompartmental analysis. *Results*. Comparing the NVP group to controls, clearance of artesunate was reduced 50% (1950 versus 2995 L/h; *P* = 0.03), resulting in a 45% increase in the AUC_0-96_ (105 versus 69 ug^*∗*^hr/L; *P* = 0.02). The half-life of dihydroartemisinin was shorter in the NVP group (1.6 versuss 3.2 h; *P* = 0.004), but other dihydroartemisinin pharmacokinetic parameters were unchanged. A lower conversion of artesunate to dihydroartemisinin was observed in the NVP group (dihydroartemisinin: artesunate AUC_0-96_ = 5.6 versuss 8.5 in NVP and control groups, respectively, *P* = 0.008). *Conclusion*. Although NVP-containing ART impacted some pharmacokinetic parameters of artesunate and dihydroartemisinin, overall exposure was similar or better in the NVP group.

## 1. Introduction

Malaria remains a disease of public health importance with an estimated 169–294 million cases in 2009, resulting in approximately 781,000 deaths [1]. Sub-Saharan Africa not only carries a high burden of the morbidity and mortality associated with malaria but also a disproportionate burden of HIV disease. An estimated 33.3 million people are living with HIV throughout the world, with more than 65% living in sub-Saharan Africa, contributing 72% of the global HIV/AIDS-related mortality in 2009 [2]. HIV and malaria comorbidity is common given the overlapping geographic areas affected by both diseases [3, 4]. The safe and effective treatment of these common coinfections is a public health priority.

As part of efforts to combat drug resistance, the World Health Organization (WHO) first recommended the use of artemisinin-based combination therapy (ACT) for malaria in 2006 [5] and upheld this recommendation in the 2010 guidelines [6]. Based on this recommendation, artesunate-amodiaquine is one of two regimens endorsed in the Nigeria Malaria Treatment Policy since 2005 while the other is artemether-lumefantrine [7]. Both regimens are used at all levels of care in Nigeria, from home management to tertiary care facilities. Artemisinin resistance has emerged since the initial 2006 WHO malaria treatment guidelines [5, 6], emphasizing the need for vigilant use of these essential medications.

Many HIV-infected patients receiving combination antiretroviral therapy (ART) will inevitably require concomitant use of an ACT in many regions of the world. The complex pharmacology of both ACTs and antiretroviral drugs lends concern to the safe and effective use of these agents in combination. Artesunate is primarily metabolized via esterase-mediated hydrolysis, but also by the cytochrome p450 (CYP) 2A6 isoenzyme, to the active metabolite dihydroartemisinin (DHA) [8]. DHA is subsequently metabolized via uridine diphosphate glucuronosyltransferases (UGTs) 1A9 and 2B7, and excreted in the bile [9]. Generally, both artesunate and DHA are moderately to highly protein bound with an elimination half-life of less than one hour, although DHA has a marginally longer half-life than artesunate [10–12]. Artesunate and DHA both possess antimalarial activity, with DHA being the more potent of the two. Therefore, any drug interaction assessment of artesunate must consider both artesunate and DHA.

Nevirapine (NVP), a nonnucleoside reverse transcriptase inhibitor (NNRTI), is a component of most first-line ART regimens in sub-Saharan Africa. NVP is metabolized via CYP 3A4 and CYP 2B6 and induces its own metabolism via induction of CYP 3A4 and perhaps 2B6 [13–15]. The potential for pharmacokinetic interactions between ACT and NVP has not been fully explored. Unfavourable pharmacokinetic drug interactions may lead to supratherapeutic antimalarial or antiretroviral concentrations, resulting in toxicity or, conversely, subtherapeutic concentrations resulting in treatment failure or drug resistance. There is also the potential for positive pharmacologic interactions, which may be beneficial to patients with malaria and HIV co-infection. In either instance, there is an urgent need to investigate the potential drug interactions resulting from the coadministration of NVP and ACT. Therefore, the primary objective of this study was to explore the pharmacokinetic interactions between NVP and artesunate taken in combination with amodiaquine in asymptomatic HIV-infected Nigerian adults by evaluating the disposition kinetics of artesunate and DHA in the presence and absence of steady-state NVP.

## 2. Materials and Methods

Patient recruitment, care, and follow up took place at the University College Hospital, Ibadan, Nigeria. The University of Ibadan/University College Hospital Institutional Review Board approved this study, and all patients provided written, informed consent. Eligible subjects had confirmed HIV-1 infection, were over 18 years of age, and had adequate renal and hepatic function, defined as serum creatinine <1.5 mg/dL and alanine transaminase and aspartate transaminase <1.5 times the upper limit of normal, respectively. Subjects were recruited into two groups: (1) NVP group and (2) control group. Subjects in the NVP group were on the same ART, consisting of lamivudine (3TC) 150 mg, zidovudine (AZT) 300 mg, and NVP 200 mg taken twice a day for a minimum period of 8 weeks prior to study enrolment, while all patients in the control group were not yet receiving antiretroviral therapy. Pregnant women, patients with known intolerance to study drugs, and patients who used artemisinin derivatives or other drugs known to induce or inhibit the CYP enzyme system in the preceding four weeks were excluded from the study. All the participants were in a good state of health, with leukocyte, haemoglobin, and hematocrit values within normal limits, no gastrointestinal symptoms or other physical complaints as judged by their primary physician. Patients remained on their current ART (NVP group) or were ART-free (control group) for the duration of the study.

A comprehensive history was obtained from individuals who met the inclusion criteria, including duration of HIV infection, drug history, and past medical history. Targeted physical examination included pulse, blood pressure, weight and height measurements. A capillary blood sample was collected via finger prick for malaria screening; however the results of the screening did not preclude study participation and one patient in each study group was found to be positive for malaria. In addition, about 10 mls of venous blood was drawn to determine baseline renal and hepatic function as well as pretreatment artesunate pharmacokinetics. These pretreatment samples were used to confirm no patients had detectable artesunate concentrations at the time of initiating the study, but were not used in the pharmacokinetic analysis. Subsequently, each participant received oral artesunate 200 mg and amodiaquine 600 mg daily for three days. Samples for the determination of artesunate plasma concentrations were collected according to the following schedule: predose (0 h) on the third day, and 0.5 h, 1 h, 1.5 h, 2 h, 3 h, 4 h, 6 h, 8 h, 10 h, 12 h, 24 h, 48 h, 72 h, and 96 h after the 3rd and last dose of artesunate-amodiaquine. All the samples were immediately centrifuged, separated, stored in a −80°C freezer, and were later batch shipped on dry ice to the Clinical Pharmacology Laboratory at the Mahidol-Oxford Tropical Medicine Research Unit in Thailand for artesunate and DHA quantification. A repeat sample to assess renal and liver functions was taken on day 7 of the study, or 96 h following the last dose of the artesunate-amodiaquine.

### 2.1. Artesunate and Dihydroartemisinin Quantification

The plasma concentrations of artesunate and DHA were determined using solid-phase extraction and liquid chromatography-tandem mass spectrometry on an API 5000 triple-quadruple mass spectrometer (Applied Biosystems/MDS SCIEX, Foster City, CA) with a TurboV ionization source operated in the positive ion mode [16]. Stable isotope-labeled artesunate and stable isotope-labeled DHA were used as internal standards. Total assay coefficients of variation during analysis of all batches for artesunate and DHA were <6% at all quality control levels (5.87, 117, 1880 ng/mL for DHA, and 2.90, 51.7, 546 ng/mL for artesunate). The lower limits of quantification (LLOQ) for artesunate and DHA were set at 1.2 and 2.0 ng/mL, respectively.

### 2.2. Pharmacokinetic and Statistical Analyses

Demographic data were compared between the group on NVP and the control group using epi-info version 6. Proportions were compared using *χ* ^2^ with Yates' correction or Fisher's exact tests. Normally distributed, continuous data were compared by Student's *t*-test for independent groups. Standard non-compartmental methods were used to estimate pharmacokinetic parameters. These parameters included the area under the concentration-time curve (AUC_0–96_), maximum plasma concentration (*C*_max⁡_), time of *C*_max⁡_ (*T*_max⁡_), elimination half-life (*T*_1/2_), apparent distribution volume (Vd/F), and apparent oral clearance (CL_ss_/F), where F is the oral bioavailability. The maximum plasma concentration (*C*_max⁡_) and *T*_max⁡_ were estimated by inspection of the raw data. Continuous variables were presented as the mean (standard deviation) for subjects who participated in the study in each group, except that the discontinuous variable, *T*_max⁡_, was given as median (range). The Kruskal-Wallis test was used to determine *P* values for all parameters except *T*_max⁡_, where the Wilcoxon test was used, and the ratio of DHA to artesunate AUC_0–96_ where the Mann Whitney *U* test was most appropriate.

## 3. Results

### 3.1. Demographic and Clinical Characteristics

A total of 21 adult Nigerians consented and completed the study per protocol: 10 participants were included in the NVP group (7 (70%) female), while the other 11 constituted the control group (8 (73%) female). The NVP group was relatively older than the control group (mean (SD): 39.7 (13.5) versus 35.8 (6.4) years, respectively, *P* = 0.008), but the mean body mass index was similar between groups (23.2 (2.9) versus 22.8 (4.6) kg/m^2^; *P* = 0.6). The NVP group received NVP containing ART for a mean duration of 1.65 years with shortest duration of exposure being 6 months; thus all participants in the NVP group were at steady-state NVP exposure. The mean CD4 counts for the NVP and control groups were 415 (229) cells/mm^3^ and 438 (219) cells/mm^3^, respectively (*P* = 0.82). None of the participants smoked or consumed heavy alcohol. Artesunate-amodiaquine was well tolerated in all participants, with the only reported side effect being moderate-to-severe weakness: 2/10 (20%) in the NVP group, 1/11 (9%) in the control group, *P* = 0.93. Four individuals in the control group discontinued the study protocol due to weakness, vomiting, diarrhoea, dizziness, and anorexia. No individual in the NVP group experienced treatment-limiting adverse effects.

### 3.2. Pharmacokinetic Parameters

**Figure 1:**
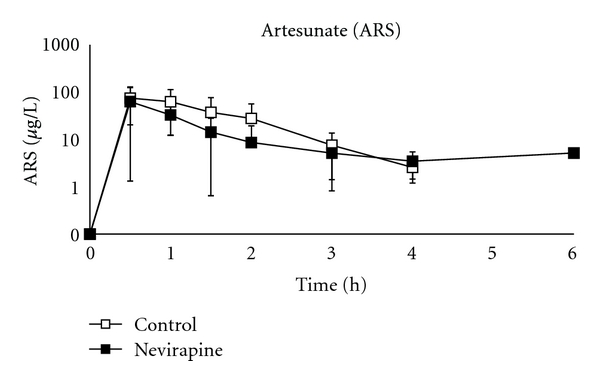
Mean plasma concentration versus time profile of artesunate (0–6 hours).

Pharmacokinetic parameters of artesunate and DHA are presented in Table 1 and Figure 1. For artesunate, the Vd/F of the NVP group was 75% smaller than that of the control group, while the Cl/F was reduced by 50% in the NVP group (*P* = 0.01 and *P* = 0.03, respectively). These changes resulted in a trend toward a lower *T*_1/2_ in the NVP group (*P* = 0.06), owing to the Vd/F and Cl/F, while the slower Cl/F in the NPV-group resulted in a 45% increase in the artesunate AUC_0–96_ (*P* = 0.02). No statistically significant differences were seen with other artesunate parameters.

**Figure 2:**
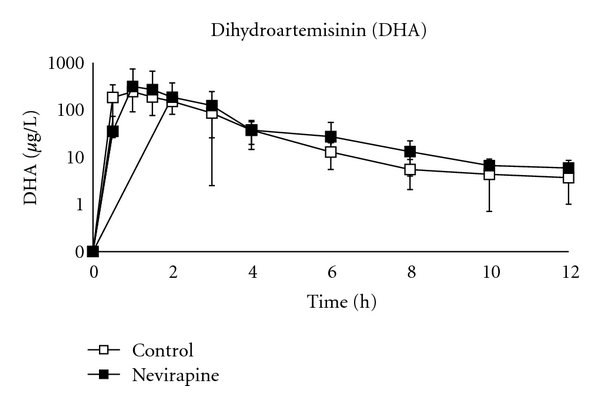
Mean plasma concentration versus time profile of dihydroartemisinin (0–12 hours).

The DHA pharmacokinetic parameters are presented in Table 1 and Figure 2. While the Vd/F of DHA in patients on NVP was 55% lower than control patients (*P* = 0.04), the Cl/F of DHA was not different between groups (*P* = 0.53). This resulted in an overall shorter *T*_1/2_ in the NVP group (*P* = 0.004), but no significant change in the overall exposure to DHA (*P* = 0.19). The ratio of DHA to artesunate, based on a comparison of AUC_0–96_, was markedly lower in the NVP group compared to the control group; (median (intraquartile range)) 5.6 (4.4–6.6) versus 8.5 (7.2–18.5), *P* = 0.008.

## 4. Discussion

To our knowledge, this study represents the first investigation of the disposition kinetics of artesunate and DHA in HIV-infected adults with and without NVP containing ART. Overall, despite a shorter *T*_1/2_ for both artesunate and DHA, we found an increase in overall exposure (AUC_0–96_) of artesunate in patients receiving NVP compared to those not on ART (105 versus 69 ug ∗ L/hr; respectively; *P* = 0.02) and no difference in the overall exposure to DHA. While the clinical relevance of these results remains unclear, it is noteworthy that the half-life of DHA was significantly shorter when given with NVP, and the conversion of artesunate to DHA was lower in the NVP group. It is possible that a negative impact of NVP on the disposition kinetics of artesunate and DHA may be detected in larger studies. This demands an observant approach to malaria therapy in individuals on NVP containing ART until further investigation into the impact of this interaction can be performed.

Given the metabolic pathways of artesunate (hydrolysis and CYP2A6) and DHA (UGT 1A9 and 2B7), the observed impact on artesunate and DHA pharmacokinetics is unexpected. Nevirapine is well known for decreasing exposure to coadministered medications due to induction of the CYP3A4 and 2B6 isoenzymes [13–15]. Interestingly, one other ACT-nevirapine interaction study described an *in vivo* pharmacokinetic interaction where NVP both increased and decreased exposure to the coadministered ACT [17]. Kredo and colleagues described the interaction between NVP and artemether-lumefantrine in HIV-infected subjects in South Africa in which lumefantrine Day 7 concentrations and AUC_0–inf_ were increased in patients on NVP compared to HIV-infected controls [17]. These directional changes seen with the lumefantrine parameters when combined with NVP are similar to our artesunate results, despite different metabolic pathways of the two antimalarial agents. Contrary to our artemisinin pharmacokinetic results, Kredo and colleagues found that the artemether and DHA AUC_0–inf_ were lower in the NVP group compared to controls [17]. Notably, different CYP enzyme pathways metabolize artesunate (CYP2A6) and artemether (CYP3A4), which may account for the difference in artemisinin pharmacokinetic findings observed in our study of artesunate compared to the results of artemether plus NVP. Although the current study was not designed to evaluate the mechanism of this interaction, our observation of a lower conversion of artesunate to DHA in the NVP group (DHA: artesunate AUC_0–96_ = 5.6 versus 8.5 in NVP and control groups, respectively, *P* = 0.008) is noteworthy. Further investigation into the underlying mechanism of this unexpected change is warranted.

The rate of malaria parasite clearance has been associated with the overall exposure to both parent drug and DHA for other artemisinins [18]; hence reduction in the blood concentrations of either or both components may negatively impact on the antimalarial activity of the artemisinin therapy. Reassuringly, our findings suggest that although the T_1/2_ was shorter, the overall exposure of both artesunate and DHA was similar compared to our control group and indeed higher for artesunate. Artesunate is generally a well-tolerated medication, particularly in comparison to other nonartemisinin antimalarial medications [19]. Dizziness, nausea, vomiting, and anorexia have been reported in patients with malaria who were treated with artemisinin monotherapy [19]. However, these toxicities were typically transient and resolved after 1-2 days, raising some question as to the relationship of the toxicity to the medication versus the underlying infectious process. Given the relative safety of artesunate, the observed increase in drug exposure would not be expected to cause additional toxicity; however vigilance for excess toxicity may be warranted.

Artesunate and DHA are known to have wide interpatient variability in their pharmacokinetic parameters, and artesunate and DHA exposure are both decreased by the co-administration of amodiaquine [20]. Additionally, the pharmacology of these agents is known to be different between patients with acute malaria and healthy volunteers. DHA total exposure was shown to be approximately 2-fold higher in patients with active malaria than healthy volunteers (4,024 versus 1,763 nmol ∗ h/L) [21]. Additionally, the protein binding of DHA may change during acute malaria infection related to plasma pH and circulating *α*-1-acid glycoprotein [22]. Complicating the evaluation of these important drug interactions further, differences in antiretroviral pharmacokinetics and pharmacodynamics exist between healthy volunteers and HIV infected patients [22, 23]; therefore, it is conceivable that HIV-infection may impact antimalarial drug concentrations as well.

There are some limitations in the present study that must be considered. In addition to noncompartmental analysis of artesunate and DHA, a comodelling approach that combines the parent and metabolite is currently underway to more fully describe the pharmacokinetic implications of chronic NVP therapy on artesunate and DHA. The pharmacokinetics of concurrent amodiaquine will be also described to fully understand the impact of NVP on antimalarial treatment with artesunate-amodiaquine. Although we have accommodated for the potential impact of HIV infection on the pharmacokinetics of artesunate and DHA by evaluating this interaction in an HIV-infected population, the pharmacokinetic impact of this interaction may be different in patients with acute malarial infection.

## 5. Conclusions

In summary, in HIV-infected patients receiving NVP-containing ART, standard multidose therapy with artesunate-amodiaquine resulted in higher overall exposure to artesunate and similar overall exposure to DHA, compared to HIV-infected patients not yet receiving ART. However, the conversion of artesunate to DHA was impaired in patients receiving NVP, and the *T*_1/2_ of DHA was shorter, both raising potential concern for the overall impact of NVP on the efficacy of artesunate. The impact of NVP on the amodiaquine component of the antimalarial therapy will provide additional insight into the safety and efficacy of combining artesunate-amodiaquine and NVP.

## Figures and Tables

**Table 1:** Comparison of pharmacokinetic parameters of artesunate and dihydroartemisinin.

Artesunate	Nevirapine Group, *n* = 10	Control Group, *n* = 11	*P*-value
	Mean (SD)	Mean (SD)	
*C*_max⁡_ (*μ*g/mL)	108 (42)	71 (57)	0.15
*T*_max⁡_ (h)*	1.0 (0.5–2.0)*	1.0 (0.5–1.5)*	0.67
Cl/F (L/h)	1950 (543)	2995 (1180)	0.03
Vd/F (L)	1162 (856)	4525 (3535)	0.01
*T*_1/2_ (h)	0.4 (0.3)	1.1 (0.9)	0.06
AUC_0–96_ (*μg*∗*L*/*h*)	105 (31)	69 (26)	0.02

Dihydroartemisinin	Mean (SD)	Mean (SD)	

*C*_max⁡_ (*μ*g/ml)	298 (107)	507 (429)	0.15
*T*_max⁡_ (h)*	1.0 (0.5–3.0)*	1.5 (0.5–6.0)*	0.11
Cl/F (L/h)	1130 (425)	980 (616)	0.53
Vd/F (L)	2405 (1077)	4338 (2518)	0.04
*T*_1/2_ (h)	1.6 (0.8)	3.2 (1.4)	0.004
AUC_0–96_ (*μg*∗*L*/*h*)	603 (218)	883 (607)	0.19

*Presented as median (range).
